# The Influence of Lime Solution in Kneading Water Substitution on Cement Roughcast and Mortar Coating

**DOI:** 10.3390/ma14154174

**Published:** 2021-07-27

**Authors:** André M. Santos, Ângelo J. Costa e Silva, João M. F. Mota, João M. P. Q. Delgado, Fernando A. N. Silva, António C. Azevedo

**Affiliations:** 1 Federal Institute of Education Sciences and Technology of Pernambuco (IFPE), Recife 50740-545, Brazil; andresantos@recife.ifpe.edu.br (A.M.S.); mota.joaomanoel@gmail.com (J.M.F.M.); antonio.costaazevedo@fe.up.pt (A.C.A.); 2 Department of Civil Engineering, Catholic University of Pernambuco, Recife 50050-900, Brazil; angelo.silva@unicap.br (Â.J.C.eS.); fernando.nogueira@unicap.br (F.A.N.S.); 3 CONSTRUCT-LFC, Departamento de Engenharia Civil, Universidade do Porto, Rua Doutor Roberto Frias, 4200-465 Porto, Portugal

**Keywords:** bond strength, kneading water, lime solution, mortar, roughcast

## Abstract

The understanding of the mechanical fixation behavior of coatings is crucial for a better comprehension of the bonding systems, especially at the interface between the mortar and the substrate. Physical adherence is related, among other things, to the contents of the materials used in the roughcast and mortar coatings, due to the colloidal water penetration into the pores of the substrate. This work evaluated the influence of different lime solution additions replacing the kneading water in the preparation of roughcast and mortar coatings. Two types of substrates were investigated:ceramic bricks and concrete blocks. Three wall masonry panels were constructed, with dimensions of 220 × 180 cm^2^, one of concrete block and two of ceramic bricks, followed by the application of roughcast and mortar coating with an average thickness of 5 mm and 20 mm, respectively. Direct tensile bond strength tests were performed and the results, with a 95% confidence level, showed that substrate ceramic and treatment in the roughcast exhibited a better behavior regarding the distribution of the tensile bond strength of the tested specimens. However, no significant differences of the amount of addition used (0%, 5%, 10% and 15%) on the tensile bond strength were observed.

## 1. Introduction

A primary step to obtain an adequate behavior of mortar coating systems is the preparation of the base to assure an appropriate and efficient tensile strength of the system. The execution of a roughcast layer is one of the most used strategies used to ensure such a base because it standardizes the characteristics of the base, increases the contact area and, mainly, increases the roughness of the surface.

Carasek [1] showed that over poor bases, the bond strength of the substrate is inversely proportional to the moisture and directly proportional to cement amount. Torres et al. [2] reported the influence of substrate porosity on adhesion, based on an experimental study developed with different types of bases and mortars. High absorption substrates can result in insufficient water for the complete hydration of cement near the interface area between the substrate and the roughcast, which weakens this region (approximately 20 μm). On the other hand, substrates with low absorption can lead to water accumulation in the interface zone, increasing the porosity and contributing to the increase of the water/cement ratio, compromising its performance [3].

Stolz et al. [4] identified an even more significant influence of the rheological behavior of the mixture than on the roughness of concrete substrates in the adhesion of mixed cement mortars and hydrated lime.

Previous works have already reported the role the lime plays over the substrate adhesion mainly because it improves workability, water retention, initial and extended adhesion, in both fresh and hardened states. Furthermore, it improves the substrate strengths-to-surface abrasion, its compression and tension, and also its deformability capabilities [5,6,7,8].

Concrete blocks, in general, present lower total water absorption and higher initial absorption than the ceramic bricks. In the case of ceramic bricks, porosity is very dependent on the manufacturing characteristics, mainly its sintering temperature. Azevedo et al. [9] evidence this influence, correlating the adhesion of mortar coatings with firing temperature, with an increase of up to 50% in their behavior when the temperature increases from 750 °C to 950 °C.

The bond strength between substrate and mortar depends on many interrelated factors, such as mortar and substrate properties as well as workmanship and curing conditions [10]. The development of bonds is associated to the mortar water retention and the suction by the substrate, as both control moisture transport at the interface influencing mortar flowing and hydrates formation [11]. Thus, the interfacial moisture content affects the bond strength between brick and mortar. To further increase the adhesion in the interface, it is common to spray lime solution before the application of the roughcast or plaster mortar, as described in several studies [4,12,13,14,15,16].

This work aims to evaluate the influence of different lime solution additions in the kneading water of roughcast and plaster mortar, namely on the tensile bond strengths of mortar with ceramic brick and concrete blocks substrates.

## 2. Materials and Methods

This experimental study was designed to analyze the tensile bond strength of mortar coatings on two specific type of substrates, ceramic bricks and concrete blocks, simulating the coatings and substrates commonly used in residential buildings. The characteristics of the materials used are described in the following sections together with an overview of the experimental program.

### 2.1. Materials

#### 2.1.1. Raw Materials

**Figure 1:**
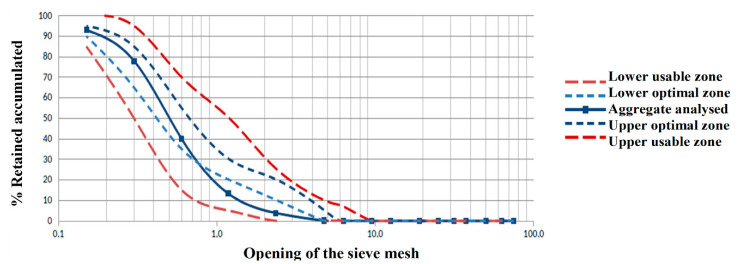
Granulometry curve of the fine aggregate used.

In this work, Portland cement composed with pozzolanic material type CP II Z-32, in accordance with the Brazilian standard NBR 16,997 [17] was used with hydrated lime, CH-I type. Table 1 and Table 2 present the physical and chemical properties of the cement and hydrated lime used, respectively. Chemical composition of cement CP II Z-32 was obtained through X-ray fluorescence (XRF) with a using Shimadzu equipment, model XRF 1800 (Shimadzu, Kyoto, Japan). The aggregate (see Table 3 and Figure 1) used was washed river silica sand (natural quartz with a maximum characteristic diameter of 2.36 mm and fineness modulus of 2.28) and the kneading water used came from a local public supply, in accordance with NBR 15900-1 [18].

#### 2.1.2. Substrate

Two types of substrates were investigated: ceramic bricks and concrete blocks. Three wall masonry panels were constructed, with dimensions of 220 × 180 cm^2^, one of concrete block and two of ceramic bricks, one of which was located outside the laboratory, simulating real conditions (ceramic bricks), and the other two were placed inside. The three study panels were erected on the same day and covered with the same materials. The following sample preparation details are given for better understanding according to the three test conditions devised for this study.

The rate of absorption (IRA) of the ceramic bricks and the concrete blocks were calculated according to ASTM C67-17 [19]. First, the relative moisture of the bricks and the blocks were removed using an oven during 48 h at a temperature varying in the range of 100 °C and 110 °C. This process was followed by a cooling phase during 4 h at room temperature dry mass of the samples. Subsequently, the bricks and blocks were immersed in a 3 mm water layer during 1 min and, after that, all water excess was removed from the contact surface and the wet mass was determined.

Compressive strength tests were performed to characterize the brick’s and block’s mechanical properties in the same position in which these elements were used to build the masonry walls. A load testing machine with a nominal capacity of 300 kN was used according to NBR 15270-1 [20] and NBR 12,118 [21].

#### 2.1.3. Mortars

**Figure 2:**
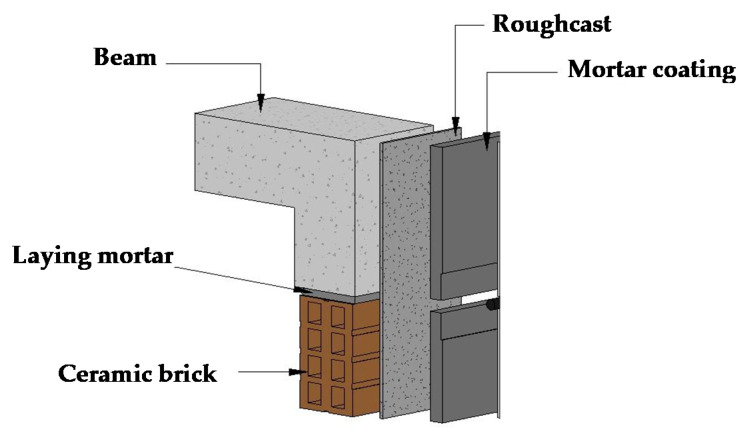
Details of the coating constitution.

To evaluate the effect of kneading water blended with different lime solution additions (0% as reference, 5%, 10% and 15%) in the preparation of roughcast and mortar coating, an experimental campaign was performed. Figure 2 exhibits the details of the mortar layers’ constitution used in the experimental campaign.

Industrialized plastering mortar with specific use for coating in field conditions was used. The kneading water used was prepared adopting the different lime additions–i.e.,: 50 g of hydrated lime for every 1000 mL of water, in the case of the 5% proportion, and 100 g and 150 g for the 10% and 15% proportions, respectively.

To define the amount of lime solution used in the mixtures, the proportion of 8.2 L for every 10 kg, in the case of roughcast samples, and 16 L of water for every 100 kg of industrialized mortar was used. Such quantities were defined as having an adequate consistency for application on the walls.

Manual spraying was used to apply mortar over the wall surfaces, followed by wet curing conditions for three consecutive days. This work was made by the same bricklayer to reduce construction errors.

**Figure 3:**
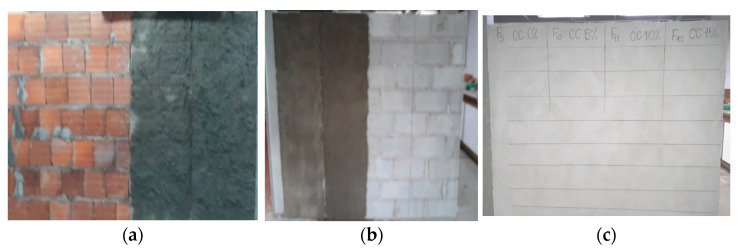
(**a**) Wall of ceramic bricks; (**b**) wall of concrete blocks, all with roughcast applied; and (**c**) wall of ceramic bricks with roughcast and mortar coating applied.

Three masonry walls were built for the development of the research, one built with concrete blocks and two others built with ceramic bricks. One of the ceramic brick walls was placed outdoors in order to simulate field conditions found in real construction sites, and the remaining wall remained inside the test laboratory. All the walls were built in a single day and their mortar coatings were also executed on this same day (see Figure 3). Roughcast and mortar coating with an average thickness of 5 mm and 20 mm was used, respectively.

The amount of kneading water was determined from the minimum amount necessary to produce plasticity identified as suitable 5 mm thick roughcast. It should be highlighted that in the roughcast layer, a reduced water–cement ratio mitigates porosity at the interface between the paste and the substrate, just as the use of lime increases the extent of adhesion [1,6,22].

The base (roughcast) was executed 24 h after erecting the walls. Curing was executed by spraying water on the roughcast for three consecutive days following its application. After this period of curing, an industrialized 20 mm mortar coating was manually applied to the wall surfaces. This work was made by the same bricklayer to reduce construction errors.

Different roughcast and mortar coating samples were produced with different additions of lime solution replacing the kneading water (0% of lime solution—reference 5%, 10%, and 15%).

The roughcast mixture used was 1:3:0.8 (cement, sand, water–cement ratio), and the mortar layer mixture used to build the masonry walls was 1:6:1.5 (cement, lime, sand, and water–cement ratio).

### 2.2. Methods

#### Bond Strength

**Figure 4:**
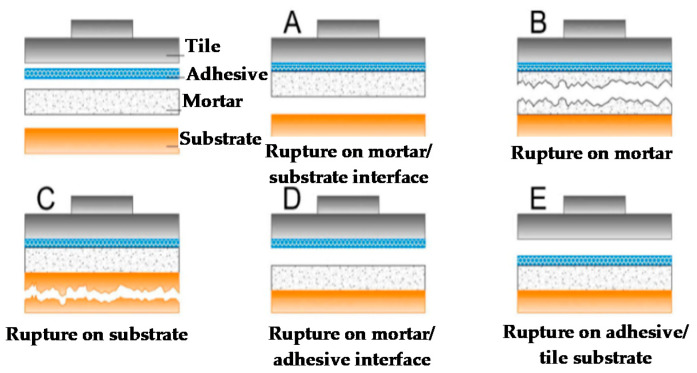
Types of rupture in the tensile bond strength tests: (**A**) Rupture on mortar/substrate interface, (**B**) Rupture on mortar, (**C**) Rupture on substrate, (**D**) Rupture on mortar/adhesive interface and (**E**) Rupture on adhesive/tile interface.

Pull-out tests were used to evaluate the mortar–substrate bond strength (18 points per sample analyzed), after 28 days and following the procedures described in NBR 13528-3 [23]. Figure 4 shows the types of rupture in the tensile bond strength tests. Special care was adopted to select the places for the pull-out tests on the wall surface to avoid undesirable failures outside the substrate that could cause inaccurate results.

This test method consists of applying the mortar, using PVC plastic ring molds (50 mm internal diameter and 100 mm in height) with a thickness of 20 ± 2 mm on a porous ceramic brick and concrete block surface. Twenty-eight days after the mortar application, metallic pull-head plates were glued with a polyester glue to the mortar surface. After 24 h, the pull-off test was performed with a dynamometer.

Special precautions were adopted in the masonry joints to avoid any influence on the test. Highly adhesive epoxy (Sikadur 31 by Sika) was used. Metal dollies were glued to the test unit of each coating with quick-drying plastic glue. This glue provides greater force than the coating can withstand, guaranteeing the dolly’s adhesion to the coating for bonding the circular dolly for the tensile strength test of the coatings, cutting the mortar with a cutting disc (cup saw).

## 3. Results and Discussion

### 3.1. Material Characterization Tests

**Figure 5:**
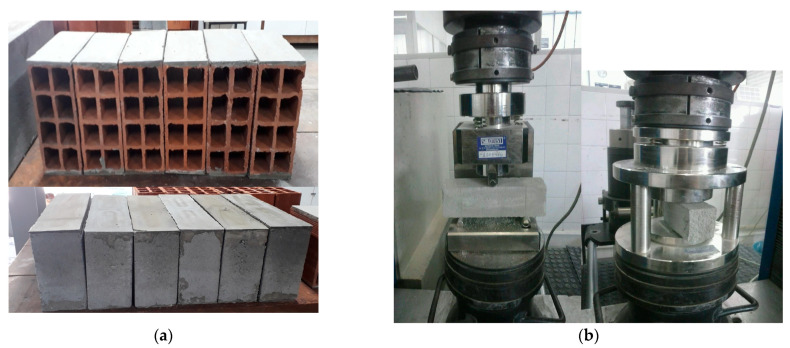
(**a**) Ceramic and concrete blocks used and (**b**) tensile test in flexion and compression test on mortar prims.

The substrates used in this experimental campaign are presented in Table 4 and Figure 5. The compressive strength was determined according to NBR 15270-1 [20] and NBR 12118 [21], for ceramic and concrete blocks, respectively. For each substrate, 19 samples were tested, 13 for compressive strength, and the others for geometric characterization and determination of physical indices (Total Absorption and Initial Rate of Absorption, IRA).

According to Paes [24] and Green [25], there is no consensus regarding the relationship between IRA and adhesion. Some of the criticisms made to this determination process are that the time involved in the test performance is relatively short for constituting a water transport mechanism since the capillary forces continue to act for a more extended period. Likewise, he mentions that the referred to process occurs in “free” water and not in “restricted” water, of which fresh mortar is comprised.

The fact that this absorption of free water (IRA) is not impeded by various forces acting on mortar is also noted. These forces are capillary forces, physical adsorption by the mortar components, and, at a later stage, the chemical bond of the water due to the evolution in the hydration of the binder (cement). In reality, substrate and mortar must be considered two independent pore systems, as the interaction between these systems determines water flow.

The mortars using in the first (roughcast) and second layer (plaster mortar), before the finishing coating, were analyzed mechanically (compressive and tensile strength) in the hardened state, and according to the NBR 13,279 [26] standard. Several prismatic samples of mortar with different percentages of lime solution instead of kneading water were prepared and tested, and the results are presented in Table 5. These results show that the compressive and tensile strength increase with the increase of lime solution on the kneading water.

### 3.2. Tensile Bond Strength Tests

The sample analyzed comprises 288 bond tensile strength test results–144 samples made over concrete substrate and 144 over ceramic substrate. To test the normality of the residual distribution and the homogeneity of variances of the tensile bond strength in the sample studied, statistical tests were performed. These tests are important because they provide essential information for deciding whether a parametric statistical analysis can be used on the data from the sample space.

Levene’s test was used to check the homogeneity of variance and a Kolmogorov–Smirnov test with the Lilliefors correction together with a Shapiro–Wilk test were used to check the normality of the residuals. Results of these statistical tests are presented in Table 6 and Table 7.

Significance levels for both tests were less than 0.005, which means that the tensile bond strength residuals do not follow a normal distribution and their variances are homogeneous. Based on these results, non-parametric tests, also known as free distribution tests, were performed. The term “free distribution” is commonly used to indicate that these tests are applicable regardless of the form of the distribution, or that they are valid for one or more broad spectrums of distribution.

**Figure 6:**
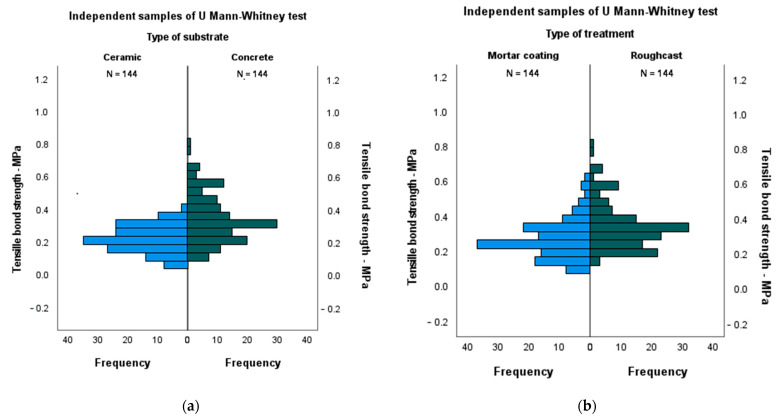
U Mann–Whitney test for (**a**) the type of substrate and (**b**) for the type of treatment.

The Mann–Whitney with two independent sample tests were performed (see Figure 6), in order to analyze the influence of each independent variable investigated, i.e., type of substrate (bricks or concrete blocks), type of treatment (roughcast or mortar) and the addition percentage, on the tensile bond strength of the tested specimens. This test is often used when a comparison of differences between two independent groups is desired and when the dependent variable is either ordinal or continuous, but not normally distributed.

It should be highlighted that for both types of substrates and treatments, the experimental campaign included two distinct groups: concrete or ceramic bricks and roughcast or mortar coating treatments. Table 8 shows the results obtained for the influence of the type of substrate and treatment, on the tensile bond strength, respectively. Since for both dependent variables investigated *p* values were less than 0.05 (*p* < 0.001), one can conclude that both studied substrates and types of treatment used influenced the values of tensile bond strength of the specimens. Figure 4 shows the U Mann–Whitney test for (a) the type of substrate and (b) for the type of treatment from where one can observe that the substrate ceramic and treatment in the roughcast exhibited a better behavior regarding the distribution of the tensile bond strength of the tested specimens.

In order to analyze the influence of the amount of addition used and because the experimental campaign considered four groups in this independent variable: no addition, 5% of addition, 10% of addition and 15% of addition, the Kruskal–Wallis test was performed. This test is a rank-based nonparametric test that is often used to verify if there are statistically significant differences between two or more groups of an independent variable. It is an extension of the Mann–Whitney U test that allows one to compare more than two independent groups over a dependent variable. Table 9 presents the results of this test and it is possible to observe that the significance level was higher than 0.05, which means than there was no influence of the amount of addition used on the tensile bond strength. In other words, the distribution of the tensile strength is the same across the categories of the amount of addition groups and none of the studied additions (0%, 5%, 10% and 15%) showed any statistically significant influence on the respective tensile bond strength values. 

**Figure 7:**
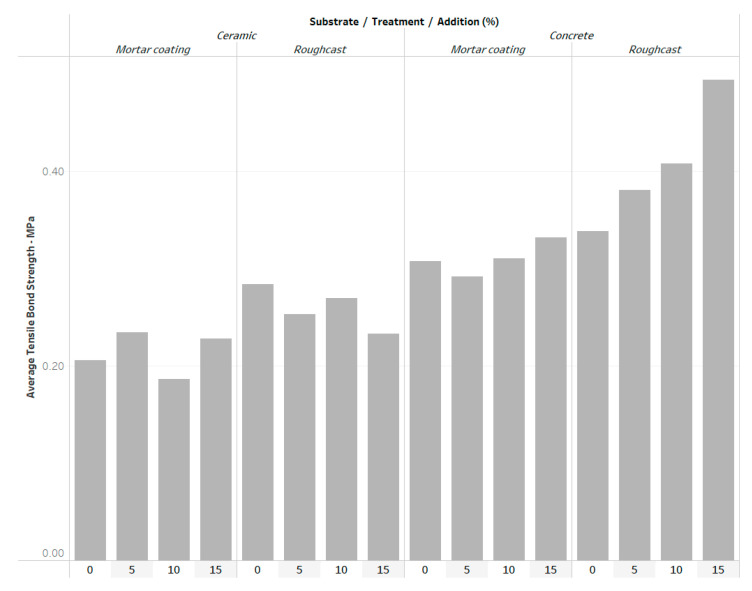
Average tensile bond strength categorized by lime solution addition.

Despite this result, it was possible to observe a qualitative trend that the best performance, in terms of tensile bond strength, was obtained for the concrete substrate with 15% of lime solution addition in the roughcast (Figure 7).

## 4. Conclusions

This research work evaluated the influence of different lime solution contents (0%-reference, 5%, 10% and 15% in relation to the amount of water used) replacing the kneading water in the preparation of roughcast and plaster mortar, for two types of substrates and two type of treatments, on the tensile bond strength of tested specimens.

The distribution of the tensile strength was shown to be equivalent across all categories of lime solution addition studied and none of the studied additions (0%, 5%, 10% and 15%) showed any statistically significant influence on the respective tensile bond strength values. Despite this result, it was possible to observe a qualitative trend that the best performance, in terms of tensile bond strength, was observed for the concrete substrate with 15% of lime solution addition in the roughcast.

The highest bond strength values were obtained for roughcast applied on concrete block bases, which is explained by the increased initial rate of absorption (IRA). This increased absorption favors the flow of the paste present in the mortar into the substrate pores, increasing the local network of the ettringite and C–S–H crystals (micro-anchorage-active pores principle). Another explanation is the greater surface roughness, which makes this material more sensitive to the penetration and accumulation of the lime solution between its interstices.

## Figures and Tables

**Table 1:** Physical and chemical properties of the cement CP II Z-32.

**Physical Characterization**	Apparent Density (g/cm^3^)		1.2 g/cm^3^
	Specific Mass (g/cm^3^)		3.09 g/cm^3^
	Compressive strength	28 days (MPa)	39.5
**Chemical Characterization (%)**	Potential composition of the clinked	3CaOSiO_2_, C_3_S	20–70
		2CaOSiO_2_, C_2_S	10–60
		3CaOAl_2_O_3_, C_3_A	1–15
		4CaOAl_2_O_3_Fe_2_O_3_, C_4_AF	5–15
	SiO_2_–silicon dioxide		22.94
	CaO–calcium oxide		57.61
	MgO–magnesium oxide		3.99
	Al_2_O_3_–aluminum oxide		5.60
	Fe_2_O_3_–iron oxide		3.99
	P_2_O_5_–phosphorous pentoxide		0.37
	K_2_O–potassium oxide		1.68
	CaO/free lime		0–2
	MgO/SO_3_		0–6

**Table 2:** Hydrated limestone description.

Composition	Limestone
CO_2_	≤2.21
Ca(OH)_2_	≥92.5%
SO_3_	≤0.05
Mg(OH)	≤5.0%
SiO_2_	≤1.3%
Relative Humidity	≤2.0%
Apparent Density	0.56 g/cm^3^

**Table 3:** Fine aggregate description.

**Unit Mass**	**1.3 g/cm^3^**
Fineness module	2.28
Maximum diameter	2.36 mm
Powder content	2.38%

**Table 4:** Characteristics of the substrate used.

Base	Dimension (mm)	Total Absorption (%)	Compressive Strength (MPa)	Initial Rate of Absorption-IRA (g/cm^2^min)
Ceramic brick	90 × 190 × 190	14.6	2.8	0.0605
Concrete block	90 × 190 × 390	6.4	4.0	0.0845

**Table 5:** Hardened mortar prisms with kneading water replaced by different percentages of lime solution.

Mortar Specimens	Tensile Strength (MPa)		Compressive Strength (MPa)	
M_0%	0.79	0.86 ± 0.09	1.49	1.98 ± 0.32
	0.99		1.73	
	0.79		2.22	
	0.80		2.43	
	0.95		2.26	
	--		1.75	
M_5%	1.07	1.11 ± 0.04	2.28	2.40 ± 0.11
	1.08		2.36	
	1.19		2.55	
	1.12		2.55	
	1.08		2.43	
	--		2.21	
M_10%	1.37	1.34 ± 0.06	3.77	3.23 ± 0.19
	1.25		3.13	
	1.39		3.19	
	1.28		2.95	
	1.39		3.27	
	--		3.06	
M_15%	1.28	1.40 ± 0.11	4.34	3.88 ± 0.28
	1.59		3.73	
	1.33		3.97	
	1.49		3.84	
	1.31		3.22	
	--		4.18	

**Table 6:** Levene’s error variance equality test.

		Levene Statistic	df1	df2	Sig.
Tensile bond strength residuals ^a^	Based on average	3.910	15	272	<0.001
	Based on median	2.752	15	272	<0.010
	Based on median with gl adjusted	2.752	15	172.155	<0.011
	Based on trimmed average	3.701	15	272	<0.001

Tests the null hypothesis that the variance of the error of the dependent variable is the same between groups. ^a^ Dependent variable: residual for tensile bond strength.

**Table 7:** Normality tests of tensile bond strength residuals.

	Kolmogorov-Smirnov ^a^			Shapiro-Wilk		
	Statistic	Gl	Sig.	Statistic	gl	Sig.
Tensile bond strength residuals	0.057	288	<0.023	0.985	288	<0.004

^a^ Lilliefors’ Correlation significance.

**Table 8:** Mann–Whitney test statistic result for the influence of the type of substrate and type of treatment on the tensile bond strength.

	Type of Substrate (Concrete or Ceramic)	Type of Treatment (Roughcast or Mortar Coating)
	Tensile bond strength	Tensile bond strength
Mann–Whitney, U	5224.000	7180.500
Wilcoxon, W	15,664.000	17,620.500
Z	−7.280	−4.511
Significance Sig. (2 tales)	<0.001	<0.001

**Table 9:** Kruskal–Wallis test statistic result for the influence of the amount of addition (% of lime solution) on the tensile bond strength.

	Tensile Bond Strength
Kruskal-Wallis, H	0.979
Df	3
Significance	0.806

## Data Availability

The data that support the findings of this study are available upon request from the authors.
